# The role of synbiotic in controlling *Salmonella* infection in broilers

**DOI:** 10.1038/s41598-026-47199-x

**Published:** 2026-04-17

**Authors:** Atef A. Salim, Nesreen A. Mohamed, Ghada A. El-Gammal, Rania I. El-Meslemany, Nessreen F. Anwar

**Affiliations:** 1 Poultry Diseases Unit, Kafrelsheikh Regional Laboratory, Agricultural Research Center (ARC), Animal Health Research Institute, Giza, Egypt; 2 Biochemistry, Nutritional Deficiency Diseases and Toxicology Unit, Kafrelsheikh Regional Laboratory, Agricultural Research Center (ARC), Animal Health Research Institute, Giza, Egypt; 3 Bacteriology Unit, Kafrelsheikh Regional Laboratory, Agricultural Research Center (ARC), Animal Health Research Institute, Giza, Egypt; 4 Bacteriology, Reference Laboratory for Veterinary Quality Control on Poultry Production Damanhour Branch, Agricultural Research Center (ARC), Animal Health Research Institute, Giza, Egypt; 5 Pathology Unit, Aswan Regional Laboratory, Agricultural Research Center (ARC), Animal Health Research Institute, Giza, Egypt

**Keywords:** *Salmonella*, Antibiotic resistance, Synbiotic, Lesions, Biochemical parameters, Pathological changes, Diseases, Microbiology

## Abstract

*Salmonellosis* significantly affects poultry production sector and public health, through the emergence of multi-drug resistant (MDR) strains, which require attempts to use sustainable, safe, and effective alternatives. This study was carried out over the period spanning 2024 to 2025 and involved two steps. The first step included *Salmonella* isolation and characterization from 25 diseased poultry farms from various regions of Northwest Delta, Egypt, focusing on some of their virulence genes and sensitivity to antibiotics. The second experimental step involved exploring the effectiveness of synbiotic compared with traditional antibiotics in reducing *Salmonella* colonization and their impact on poultry performance, including growth performance, immunological parameters, and pathological changes after artificial infection. The result revealed the presence of *Salmonella sp*. in 16% (40/250) of examined samples, including 3 serotypes, *Salmonella* Papuana, *Salmonella* Enteritidis, and *Salmonella* Kentucky, with different values of MAR index ranging between 0.21 and 0.43. Marked resistances were recorded against amoxicillin, lincomycin and spiramycin (100%), followed by ampicillin (75%), while the highest sensitivity was observed toward amikacin, gentamicin, cefotaxime, colistin sulfate, and streptomycin. All *Salmonella* strains contained the *invA* and *enterotoxin gene (stn)*, whereas *spvC* was detected in 75% of isolates. The study declared the impact of the supplementation of synbiotic in feed in improvement of the growth performance parameters, decreased *Salmonella* colonization, reduced severity of clinical signs and postmortem lesions relative to the other groups (untreated group and antibiotic treated group), in addition to its considerable improvement in hematological, biochemical, and immunological parameters and less pathological alterations than other groups.

## Introduction

*Salmonellosis* in broilers is a significant global issue affecting both avian health and food security, representing one of the most prevalent foodborne zoonosis worldwide^1,2^. Among the 2500 *Salmonella* serotypes, *Salmonella enterica subspecies enterica serovar* Enteritidis (*S.* Enteritidis) ranks among the most significant to humans, causing food poisoning diseases^3^. Several *Salmonella* species resulted in a variety of acute or chronic illnesses, including avian *Salmonellosis*^4^. The clinical symptoms represented are dehydration, gasping, depression, drooping wings and decreased appetite. Occasionally, blindness, joint swelling, and lameness may occur^5^.

In agriculture and livestock production the widespread resistance to conventional antibiotics has resulted in reduced efficacy, increased healthcare costs, and higher mortality rates. Therefore, it is crucial to identify and establish sustainable, safe, and potent alternatives. In addition, the use of antibiotics is linked to reduction of healthy gut flora that aids in the resistance against intestinal infections^6^. One such alternative is synbiotic, which consist of probiotics (beneficial live microorganisms) and prebiotics (non-digestible compounds that promote the probiotics growth). This synergistic combination was initially reported by Gibson and Roberfroid^7^.

Prebiotics are used in synbiotics to promote probiotic bacteria by altering the gut microbiota, boosting probiotic survival, and preventing pathogen colonization of the gut epithelium. Therefore, synbiotics supplementation results in higher benefits to the host than that obtained by prebiotics alone or probiotics^8^.

The aim of this proposal involves two steps. The first one was to insight *Salmonella* isolation and characterization from 25 diseased poultry farms from various regions of Northwest Delta, Egypt, focusing on some of their virulence genes and sensitivity to antibiotics. The second part was to explore the effectiveness of synbiotic compared with traditional antibiotics in reducing *Salmonella* colonization and their impact on poultry performance, including growth performance, immunological parameters, and pathological changes after artificial infection.

## Materials and methods

### Ethical approval

All experimental procedures were conducted in accordance with the guidelines of the National Institutes of Health for the care and use of laboratory animals and were approved by the Animal Care and Biosafety Committee of the Animal Health Research Institute (AHRI), Egypt (Approval No.: ARC-AHRI-8-26). The study was conducted and reported in compliance with the ARRIVE Guidelines 2.0. No experiments involving human subjects were performed.

### First step: prevalence and the antibiogram of *Salmonella sp.* in broiler farms

#### Sampling and isolation of *Salmonella*

Between January 2024 and April 2025, an entire number of 250 specimens from 25 diseased chicken farms of different breeds (12 Ross, 9 Avian, 2 IR, and 2 Arbor Acres) aged 1–40 days old with different clinical symptoms and postmortem lesions from diverse sectors of the Delta’s northwest (El Beheira, Kafr El Sheikh, as well as Alexandria provinces, Egypt), farms with capacities ranging from 3500 to 16,000 were gathered aseptically and delivered to the Reference Laboratory for Veterinary Quality Control on Poultry Production (RLQP), Damanhour branch, El Beheira province, Egypt for *Salmonella* detection.

The samples were collected from liver, cecum, spleen and yolk sac. *Salmonella* was isolated and identified following standard procedure’s method of^9^.

#### Serotyping of *Salmonella* isolates

*Salmonella* antisera (DENKA SEIKEN Co., Japan) were used for somatic (O) and flagellar (H) antigens assessment to identify *Salmonella* serotype^10^.

#### Antimicrobial sensitivity test

Clinical and Laboratory Standards Institute^11^ guidelines were followed to determine the sensitivity and resistance of *Salmonella* isolates using disc diffusion methods^12^. Using fourteen antibiotics (Himedia, India) belonging to nine classes, including Aminoglycosides (Gentamycin CN-10 ug; Streptomycin S-10 ug; Amikacin AK-30 ug; Neomycin N-30), Polymyxins (Colistin sulfate CL-10 ug), Amphotericols (Florfenicol FFC-30 ug), Cephalosporins (Cefotaxime CTX-30 ug), Beta lactams (Ampicillin AMP-10 ug, Amoxycillin AX-25), Tetracyclines (Tetracycline TE-30 ug, Doxycycline DO-30 ug), Sulphonamides (Trimethoprim-sulfamethoxazole COT, 25 µg), Macrolides (Spiramycin SP-100) and Lincosamides (Lincomycin L-2). The estimation of MAR (Multi Antimicrobial Resistance) index was determined according to^13^ in which we divided resistant antibiotics number by the overall number of antibiotics assessed. MAR index values more than 0.2 indicate resistance of the organism Piryaei et al.^14^.

#### Investigation of virulence determinants of *Salmonella* using polymerase chain reaction (PCR)

##### DNA extraction

Genomic DNA was extracted using the QIAamp DNA Mini Kit (Qiagen, Germany, GmbH) with minor modifications. Briefly, 200 µL of the strain suspension was mixed with 20 µL proteinase K and 200 µL lysis buffer, then incubated at 56 °C for 10 min. After incubation, 200 µL of 100% ethanol was added. The remaining washing and centrifugation steps were performed according to the manufacturer’s instructions, and DNA was finally eluted in 100 µL of the provided elution buffer.

##### Amplification of PCR

The isolates were examined for the presence of *invA*, enterotoxin gene *(stn)* and *spvC* genes. Each gene was amplified by using specific primer pairs (Metabion, Germany) and according to the PCR protocols described in (Table 1). The reaction volume25 µL containing 12.5 µL of Emerald Amp Max PCR Master Mix (Takara, Japan), 1 µL (20 pmol conc.) from each primer, 5.5 µL of nuclease-free water, and 5 µL of DNA template. Amplification was carried out using Applied Biosystems 2720 thermal cycler.

##### PCR amplicons analysis

1.5% agarose gel (Applichem, Germany) prepared in 1x TBE buffer under ambient conditions was used for separating PCR amplicons using electrophoresis at 5 V/cm. Each gel slot is loaded with 20 µL of PCR products. The fragment sizes were determined using, a 100 bp ladder (Generuler: Fermentas, Thermo, Germany). The gel was photographed by a gel documentation system (Alpha Innotech, Biometra), and the images were analyzed using dedicated computer software.

**Table 1:** Target genes, primer sequences, amplicon sizes, and condition of cycles.

Target gene	Primers sequences	Amplified segment	Primary denaturation	Amplification (35 cycles)			Final extension	Reference
				Secondary denaturation	Annealing	Extension		
*spvC*	ACC AGA GAC ATT GCC TTC C	467 bp	94 °C 5 min.	94 °C 30 s.	58 °C 40 s.	72 °C 45 s.	72 °C 10 min.	Huehn^15^
	TTC TGA TCG CCG CTA TTC G							
*invA*	GTGAAATTATCGCCACGTTCGGGCAA	284 bp	94 °C 5 min.	94 °C 30 s.	55 °C 30 s.	72 °C 30 s.	72 °C 7 min.	Oliveira et al.^16^
	TCATCGCACCGTCAAAGGAACC							
*stn*	TTG TGT CGC TAT CAC TGG CAA CC	617 bp	94 °C 5 min.	94 °C 30 s.	59 °C 40 s.	72 °C 45 s.	72 °C 10 min.	Murugkar et al.^17^
	ATT CGT AAC CCG CTC TCG TCC							

### **Second step: the experiment** (It was conducted on Kafrelsheikh regional laboratory)

#### Experimental design

For the pathogenicity of *Salmonella* Enteritidis to susceptible birds, we used 105, one day old Cobb broiler chicks. At the beginning, we collected five chicks randomly and sacrificed them for ensuring they were free from *Salmonella* infection through bacteriological examination Marcq et al.^18^. Chicks were reared in an ecologically controlled isolation facility and allowed diets Table 2 and water ad libitum. Birds were sub-grouped into four groups of 25 birds each as shown in Table 3. The vaccination program was started by the ND (New castle) vaccine at the 7th day and the Gumboro vaccine at the 14th day. Abou Zeid et al.^19^. The chicks in group 2, group 3 and group 4 (G2, G3, and G4) were challenged orally with 1 mL of 10^9^ CFU/mL of *S.* Enteritidis per bird at the 6th day of age according to Okamoto et al.^20^. The chicks in G3 were treated with synbiotic in a dose of 1 kg/ ton in their feeding diet from the 1st day upon experiment completion. While chicks in G4 were treated with Florfenicol antibiotic after 2 days of challenge (at the 9^Th^ day of age over five days continuously). Clinical symptoms, mortality rate, together with necropsy findings were monitored each day for challenged groups for the duration of the study, until 35 days old chicken.

**Table 2:** Physical and chemical composition of the basal diet.

	Starter	Grower	Finisher
Physical composition			
Ingredients %			
Ground yellow corn (8% CP)	56.52	60.13	64.72
Soya bean meal (46% CP)	32.57	28.87	24.16
Corn gluten (60% CP)	4.8	4.8	4.5
Soybean oil	1.8	2.26	3
L-Lysine (purity 99%)	0.4	0.34	0.34
DL-methionine (purity 99%)	0.15	0.12	0.11
Limestone (38% Ca)	1	0.8	0.7
Di-calcium-phosphate (22% Ca and 19% P)	1.955	1.925	1.765
Iodized sodium chloride	0.5	0.45	0.4
Vitamin and mineral premix**	0. 3	0.3	0.3
Pycnogenol	0.005	0.005	0.005
Chemical analysis			
Nutrients %			
Metabolizable energy (kcal/kg diet)	3024	3100	3196
Crude protein (%)	23	21.5	19.5
Calorie/protein ratio (*C/P*)	131.48	144.19	163.9
Lysine %	1.44	1.29	1.17
Methionine%	0.56	0.51	0.47
Calcium %	1	0.91	0.82
Available phosphorus	0.46	0.44	0.4
Sodium	0.23	0.20	0.18

Synbiotic amount in G3 was added in exchange for the same quantity of yellow corn.

*Formulated according to^21^, and Chemical analysis was performed according to^22^.

**Premix Supplied per kg of premix: *trans*-retinol(A), 12,500,000 IU; cholecalciferol(D3), 500,000 IU; α-tocopherol acetate(E), 75,000 mg; thiamine(B1), 4500 mg; riboflavin(B2), 8000 mg; pyridoxine(B6), 5000 mg; vitamin B12, 22,000 mg; pantothenic acid, 20,000 mg; folic acid, 2000 mg; biotin, 200,000 µg; Fe, 100,000 mg; Co,250 mg; Mn, 100 mg; Cu, 10,000 mg; Zn, 80,000 mg; I, 1000 mg; Se, 300 mg; Mo, 0.5 mg; Ca, 7.7%; P, 0.01%; Na, 0.18%; Ash, 97%.

**Table 3:** Experimental bird grouping and design.

Groups	Treatment	Challenge with S. Enteritidis strain
G1 Control (-ve)	Not treated	Inoculated with PBS
G2 Control (+ ve)	Not treated	Challenged
G3	Treated with symbiotic	Challenged
G4	Treated with antibiotic (florfenicol)	Challenged

#### Treatments

##### Synbiotic

Produced by B.O Pharma^®^. Aqua M-aster powder (a combination of prebiotics and probiotics). (1 kg / ton feed). Probiotics (Bacillus, lactobacillus, & Yeast). Prebiotic (Saccharomyces, Mannooligosaccharides (M.O.S.) and Fructooligosaccharides (F.O.S.), Enzyme mixture, Algae extract, Antioxidants, Amino acids, Vitamins, and Minerals).

##### Antibiotic (florfenicol)

ml / liter 48 h post infection for 5 successive days.

#### *S*. Enteritidis challenge strain

The previously isolated and identified *S.* Enteritidis underwent centrifugation at 3000 revolutions per minute for 10 min, sterile buffer saline was used to dilute the sediment. A McFarland 0.5 tube was used to adjust the inoculum to obtain 10^9^ CFU/mL Timms et al.^23^.

#### Growth performance

At the onset and the end of the experiment, broiler’s weight and feed intake were recorded for the assessment of growth metrics following Castell and Tiews^24^, and Tacon^25^. Growth parameters are initial body weight (IW), final body weight (FW), feed intake (FI), weight gain (WG), feed efficiency (FE), feed conversion ratio (FCR), and specific growth rate (SGR).

$${\text{Weight gain}}=\left( {{\text{Final body weight}} - {\text{Initial body weight}}} \right).$$

$${\text{Feed conversion ratio}}={\text{Feed intake }}\left( {\mathrm{g}} \right)/{\text{weight gain }}\left( {\mathrm{g}} \right).$$

$${\text{Feed efficiency}}={\text{weight gain}}/{\text{total feed intake}}.$$

$${\text{Specific growth rate}}={\text{Ln }}\left( {{\text{final weight}}} \right) - {\text{Ln }}\left( {{\text{initial weight}}} \right)/{\text{duration of experiment}} \times {\mathrm{1}}00$$

#### Laboratory examination

##### Enumeration of *S.* enteritidis from cecal content

To estimate the colonization of *S*. Enteritidis, randomly three chicks were taken and sacrificed from the challenged groups, at two and three weeks of age. 1 gram sample of cecal contents was homogenized to prepare serial dilutions in Buffered Peptone Water (BPW) (Himedia, India). followed by spreading 0.1 mL of each dilution on XLD media (Himedia, India). After incubation the suspected typical morphological colonies were identified biochemically then enumerated Sobotik et al.^26^.

##### Biochemical examination

***Blood collection***.

After 35 days, 5 birds/group were anesthetized intraperitoneal with Sodium pentobarbital (50 mg/kg). Blood was drawn from five birds selected randomly for sampling in each group using a 3 ml syringe from the wing vein. Then the gathered blood was split into two tubes: one involved heparin to prevent coagulation for blood analysis (hemoglobin (Hb), packed cell volume (PCV), WBC count, differential leukocyte count (DLC), phagocytic activity, and phagocytic index). While the other without anticoagulant to obtain serum which was kept at -20 °C for the biochemical evaluation of alanine aminotransferase (ALT), total protein, albumin, globulins, catalase, and superoxide dismutase (SOD).

***Hematological assessment***.

Hemoglobin (Hb), packed cell volume (PCV), white blood cell counts (WBCs), and differential leukocyte count (heterophils, lymphocytes, monocytes, eosinophils, and basophils) were determined by the method of Mafuvadze and Erlwanger^27^. Phagocytic activity and phagocytic index were evaluated following the technique of Kawahara et al.^28^.

***Biochemical assessment***.

Serum alanine aminotransferase (ALT) was measured following the method of Reitman and Frankel^29^, whereas serum total protein and albumin, as described by Doumas and Briggs^30^, globulin levels were determined by subtracting albumin from total protein. Uric acid was determined based on Metais et al.^31^. Catalase and superoxide dismutase according to Weydert and Cullen^32^.

##### Pathological examination

At the age of 35-days (the end of experiment), after the boilers were euthanized with overdose of sodium pentobarbital, 5 birds/group were slaughtered and pieces of proventriculus, intestine, liver, heart, bursa, spleen, and kidney were collected. The samples were fixed in 10% neutral buffered formalin then stained by hematoxylin and eosin stain (H&E) after possessing, according to Layton et al.^33^, and examined for pathological lesions.

### Statistical analysis

Analysis of the data was carried out statistically, applying GraphPad Prism 9 software (GraphPad Software, La Jolla, California, USA). One-way ANOVA with Tukey’s test was used to investigate the statistically validated differences across the groups. The outcomes are expressed as mean ± SE.

## Results

### First step: Prevalence and antibiogram of *Salmonella*

#### Clinical symptoms and postmortem (PM) lesions of collected samples

The infected birds showed depression, weight loss, ruffled feathers, gasping, nasal mucoid discharge, diarrhea, and pasty vent plumage. While PM lesions showed enlarged liver with necrosis, enlarged spleen, unabsorbed yolk sac in young chicks, and enteritis.

#### Prevalence of *Salmonella* among broiler chicken

The current investigation detected *Salmonella* species in four farms out of twenty-five 16% (40/250).

#### Serological identification of *Salmonella*

The study identified multiple *Salmonella* serotypes represented in *Salmonella* Papuana (*S*. Papuana) *C*1 O 6,7 H r: e, n, Z15 (*n* = 1), *Salmonella enterica subspecies enterica serovar* Enteritidis (*S.* Enteritidis) D1 O 1,9,12 H g (*n* = 2), and *Salmonella* Kentucky (*S.* Kentucky) C3 O 8,20 H i: Z6 (*n* = 1).

#### Antimicrobial susceptibility of *Salmonella* isolates

The results showed 100% resistance toward amoxycillin, lincomycin, and spiramycin, followed by ampicillin (75%), and low resistance rates for florfenicol and trimethoprim-sulfamethoxazole (25% each). All isolates were 100% sensitive to amikacin, gentamicin, cefotaxime, colistin sulfate, streptomycin, neomycin, and tetracycline (Table 4). Values of Multiple Antimicrobial Resistance index (MAR) were 0.21(*S*. Papuana), 0.21(*S.* Enteritidis), 0.28 *(S.* Enteritidis), and 0.43(*S.* Kentucky) (Table 5).

**Table 4:** Antimicrobial response profile of Salmonella isolates (no = 4).

Antibiotic family	Antibiotic	Sensitive		Intermediate		Resistant	
		NO	%	NO	%	NO	%
Aminoglycosides	Gentamycin CN	4	100	-	-	-	-
	Streptomycin S	4	100	-	-	-	-
	Amikacin AK	4	100	-	-	-	-
	Neomycin N	3	75	1	25	-	-
Polymyxins	Colistin sulfate CL	4	100	-	-	-	-
Amphotericols	Florfenicol FFC	3	75	-	-	1	25
Cephalosporin	Cefotaxime CTX	4	100	-	-	-	-
Beta lactam	Ampicillin AMP	-	-	1	25	3	75
	Amoxycillin AX	-	-	-	-	4	100
Tetracyclines	Tetracycline TE	4	100	-	-	-	-
	Doxycycline DO	3	75	1	25		
Sulphonamides	trimethoprim-sulfamethoxazole COT	3	75	-	-	1	25
Macrolide	Spiramycin SP	-	-	-	-	4	100
Lincosamide	Lincomycin L	-	-	-	-	4	100

**Table 5:** Antibiotic resistant pattern profiles of isolated *Salmonella* strains.

Salmonella serotypes	Antibiotic pattern profiles	No. of resistance antibiotics	MARI
*S*. Papuana	AX, SP, L	3	0.21
*S.* Enteritidis	AX, SP, L	3	0.21
*S.* Enteritidis	AX, SP, L, AMP	4	0.28
*S.* Kentucky	AX, SP, L, AMP, COT, FFC	6	0.43

#### PCR-based detection of virulence genes in *Salmonella*

**Fig. 1:**
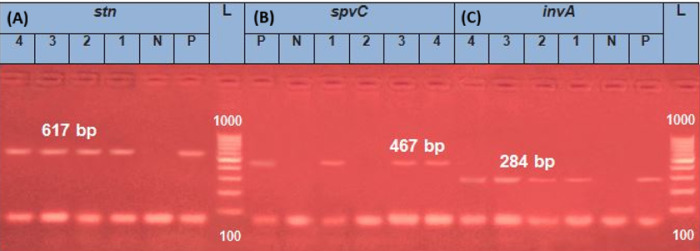
PCR-based detection of virulent genes in *Salmonella*. (**A**) PCR amplicon of *stn* gene. Lane L: 100–1000 bp molecular size marker. Lane P: positive salmonella *stn* gene at 617 bp. Lanes 1, 2, 3, 4: Positive salmonella strains for *stn* gene. (**B**) PCR amplicon of spv*C* gene. Lane Pos: Control positive salmonella *spvC* gene at 467 bp. Lanes 1, 3, 4: Positive salmonella strains for *spvC* gene, Lane 2 Negative salmonella. (**C**) PCR amplicon of *inv* gene. Lane P: positive salmonella *inv* gene at284 bp. Lanes 1, 2, 3, 4: Positive salmonella strains for *inv* gene.

All examined *Salmonella* were positive for *invA* and the enterotoxin *(stn)* genes (100%), *whereas spvC* was detected in 75% of isolates (Fig. 1).

### Second step: the experiment

#### Clinical signs of *S.* enteritidis infection

**Fig. 2:**
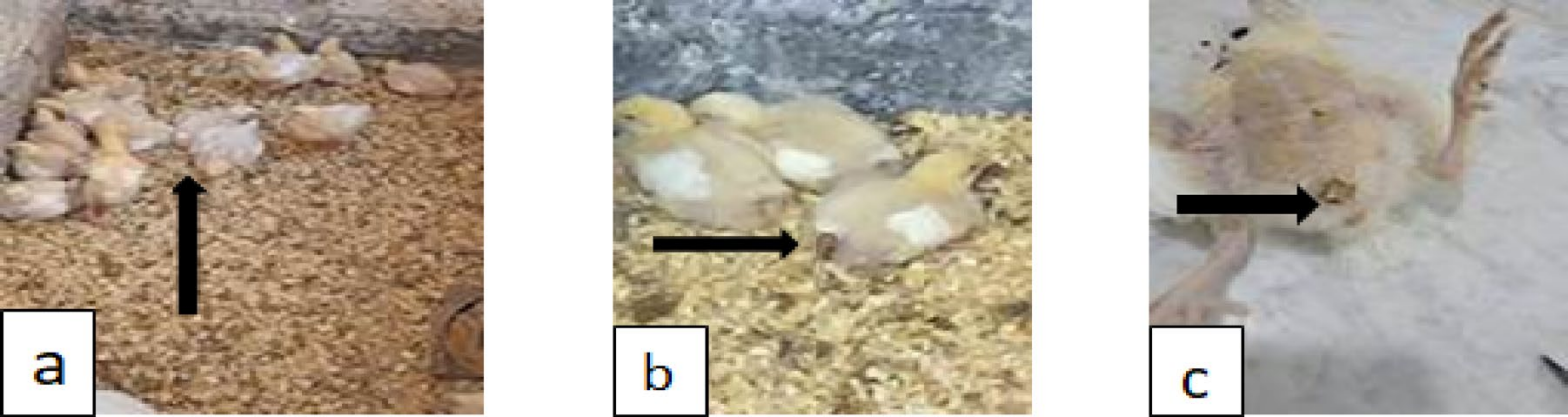
Clinical signs of *S.* Enteritidis infection. (**a**) The infected birds showed anorexia, weight reduction, and abnormal breathing patterns. (**b**,**c**) watery diarrhea with a whitish appearance.

Depression, decreased appetite, weight loss, gasping, whitish watery diarrhea, and occasionally lameness was all present in the *S.* Enteritidis*-*infected birds, but were more prominent in G2, and decreased in severity in groups 3 & 4 respectively (Fig. 2).

#### Postmortem examinations of *S.* enteritidis infection

**Fig. 3:**
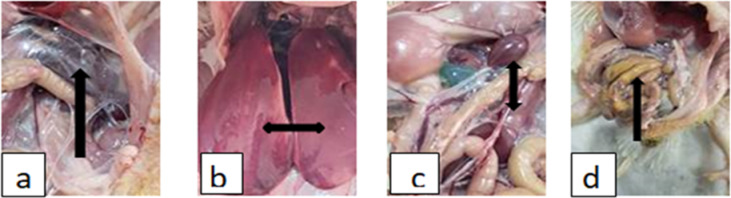
PM lesion of *S.* Enteritidis infection. (**a**) the infected birds showed airsacculitis. (**b**) sever congestion and enlargement of liver. (**c**) congestion and enlargement of spleen and congestion of kidney with deposition of urates in the ureters. (**d**) pasty cecal core.

PM lesion, showed airsacculitis, severe congestion of internal organs, liver and spleen enlargement, with deposits of urates in the ureters. These lesions were more prominent in G2, and decreased in severity in groups 3 & 4, respectively (Fig. 3).

#### Growth performance

G3 and G4 showed a marked increase in final weight, feed intake, weight gain, feed efficiency, and a marked lower feed conversion ratio, while reverse results were observed in G2 compared to G1. There was no significant difference in specific growth rate compared to G1 (Table 6).

**Table 6:** Impact of synbiotic and antibiotic supplementation on growth performance.

Growth parameters	G1	G2	G3	G4
Initial weight	44.30 ± 0.05 ^a^	44.50 ± 0.05 ^a^	45.00 ± 0.5 ^a^	44.80 ± 0.11 ^a^
Final weight	1700 ± 1.15^c^	1471 ± 0.11 ^d^	1857 ± 1.03 ^a^	1802 ± 0.28 ^b^
Feed intake	1780 ± 0.57^c^	1675 ± 1.15 ^d^	1810 ± 1.15 ^a^	1790 ± 0.57 ^b^
Wight gain	1656 ± 0.12 ^c^	1426 ± 1.15 ^d^	1812 ± 0.34. ^a^	1757 ± 0.99 ^b^
Feed efficiency	0.93 ± 0.01 ^c^	0.85 ± 0.01^d^	1.00 ± 0.02 ^**a**^	0.98 ± 0.01 ^b^
Feed conversion ratio	1.07 ± 0.01 ^b^	1.17 ± 0.01 ^a^	0.98 ± 0.01 ^d^	1.01 ± 0.01 ^c^
Specific growth rate	4.52 ± 0.01 ^a^	4.40 ± 0.11 ^a^	4.62 ± 0.01 ^a^	4.58 ± 0.01 ^a^
Mortality rate	0%	30%	20%	25%

Values with different letters in each row are significantly different at (*P* < 0.05), Values are mean ± S.E.

#### *Salmonella* colonization

Cecal colonization of *S.* Enteritidis was evaluated at 7- and 14-days post infection (14 and 21 days old); At 14 days old, the mean of *S.* Enteritidis cecal count of positive control group G2 was (3.9 × 10^6^), higher than the treated groups (G3 and G4). All examined chicks in the G3 showed lower colonization with a mean (4.3 × 10^5^), while in the G4 examined chicks showed colonization with mean (9.1 × 10^5^). At 21days old, G2 showed no decrease in colonization while G3&G4 showed a considerable decrease in cecal colonization (Table 7).

**Table 7:** Results of *S.* Enteritidis colonization (CFU/g) for cecal content.

Days-Old	G 2	G 3	G 4
14 days old	3.9 × 10^6^	4.3 × 10^5^	9.1 × 10^5^
21 days old	3.8 × 10^6^	1.4 × 10^5^	1.2 × 10^5^

#### Hematological and biochemical findings

##### Hematological findings

G2 showed a significant decline in Hb and in PCV while G3 exhibited a significant increase in them compared to G1. WBCs showed a marked rise in G2 and G3, while lymphocytes showed a significant increase in G3 and a marked decrease in G2 compared to G1. Monocytes, eosinophils, and basophils showed no significant difference between different groups, and heterophils showed only a significant increase in G2 compared with G1 (Table 8).

**Table 8:** Results of hematological parameters.

hematological parameters	G1	G2	G3	G4
Hb (g/dl)	10.33 ± 0.02^**b**^	8.60 ± 0.12^**c**^	10.80 ± 0.06^**a**^	10.36 ± 0.01^**b**^
PCV%	30.23 ± 0.02 ^**b**^	29.13 ± 0.08^**c**^	30.70 ± 0.12 ^**a**^	30.33 ± 0.01^b^
WBCs (10^3^/µl)	20.00 ± 0.00 ^**b**^	30.00 ± 0.00 ^**a**^	30.00 ± 0.00^**a**^	25.00 ± 0.57^**b**^
Heterophil %	36.00 ± 0.57 ^**b**^	40.00 ± 0.57^**a**^	34.00 ± 0.57^**b**^	35.00 ± 1.15^**b**^
Lymphocyte %	52.88 ± 0.01 ^**b**^	46.67 ± 0.01 ^**c**^	59.00 ± 1.15 ^**a**^	54.00 ± 0.66^**b**^
Monocyte %	6.66 ± 0.33 ^**a**^	5.66 ± 0.33 ^**a**^	7.33 ± 0.33 ^**a**^	6.00 ± 0.57 ^a^
Eosinophil %	4.00 ± 0.57 ^a^	3.00 ± 0.57 ^a^	4.00 ± 0.57 ^a^	4.00 ± 0.57 ^a^
Basophil %	1.00 ± 0.57 ^**a**^	0.66 ± 0.33 ^**a**^	1.33 ± 0.33 ^**a**^	1.00 ± 0.57 ^**a**^

Values with different letters in each row are significantly different at (*P* < 0.05). Values are mean ± S.E.

##### Phagocytic activity and phagocytic index

G3 and G4 showed a significant increase in phagocytic activity and phagocytic index, whereas a marked decline was found in G2 compared to G1 (Table 9).

**Table 9:** Results of phagocytic activity and phagocytic index.

Phagocytosis	G1	G2	G3	G4
Phagocytic activity %	15.9 ± 0.06 ^**c**^	9.96 ± 0.01 ^**d**^	20.41 ± 0.01 ^**a**^	17.21 ± 0.01 ^**b**^
Phagocytic index	1.19 ± 0.01 ^**c**^	0.29 ± 0.02 ^**d**^	5.09 ± 0.01 ^**a**^	4.10 ± 0.05 ^**b**^

Values with different letters in each row are significantly different at (*P* < 0.05), Values are mean ± S.E.

##### Serum biochemical parameters

G2 showed a significant rise in ALT and a marked decline in total protein, albumin, and globulins, while a notable elevation was observed in albumin and globulins in G3 compared to G1. G2 exhibited a significant increase in uric acid, while G3 displayed a marked increase compared with G1. A significant increase in catalase and superoxide dismutase was found in G3 and G4, while reverse result was in G2 compared to G1 (Table 10).

**Table 10:** Results of biochemical parameters.

Biochemical index	G1	G2	G3	G4
ALT(IU/L)	15.33 ± 0.02^**b**^	17.33 ± 0.33^**a**^	15.00 ± 0.57^b^	15.22 ± 0.01^b^
Total protein(g/dl)	2.28 ± 0.01 ^**b**^	2.01 ± 0.01 ^**c**^	2.37 ± 0.01^**a**^	2.34 ± 0.02^**b**^
Albumin(g/dl)	1.59 ± 0.01 ^**b**^	1.43 ± 0.01 ^**c**^	1.80 ± 0.06^**a**^	1.61 ± 0.01^**b**^
Globulins(g/dl)	0.47 ± 0.02 ^**c**^	0.34 ± 0.02 ^**d**^	0.75 ± 0.02 ^**a**^	0.65 ± 0.02 ^**b**^
Uric acid(mg/dl)	6.00 ± 0.57 ^**b**^	8.39 ± 0.02^**a**^	5.9 ± 0.11 ^**b**^	6.00 ± 0.57 ^**b**^
Catalase (U/ml)	55.28 ± 0.01 ^**c**^	40.19 ± 0.02^**d**^	70.39±.04^**a**^	65.76 ± 0.02^b^
SOD(U/ml)	36.90 ± 0.02 ^**c**^	27.50 ± 0.12^**d**^	45.70 ± 0.12^**a**^	40.00±.057^**b**^

Values with different letters in each row are significantly different at (*P* < 0.05). Values are mean ± S.E.

#### Histopathological findings

**Fig. 4:**
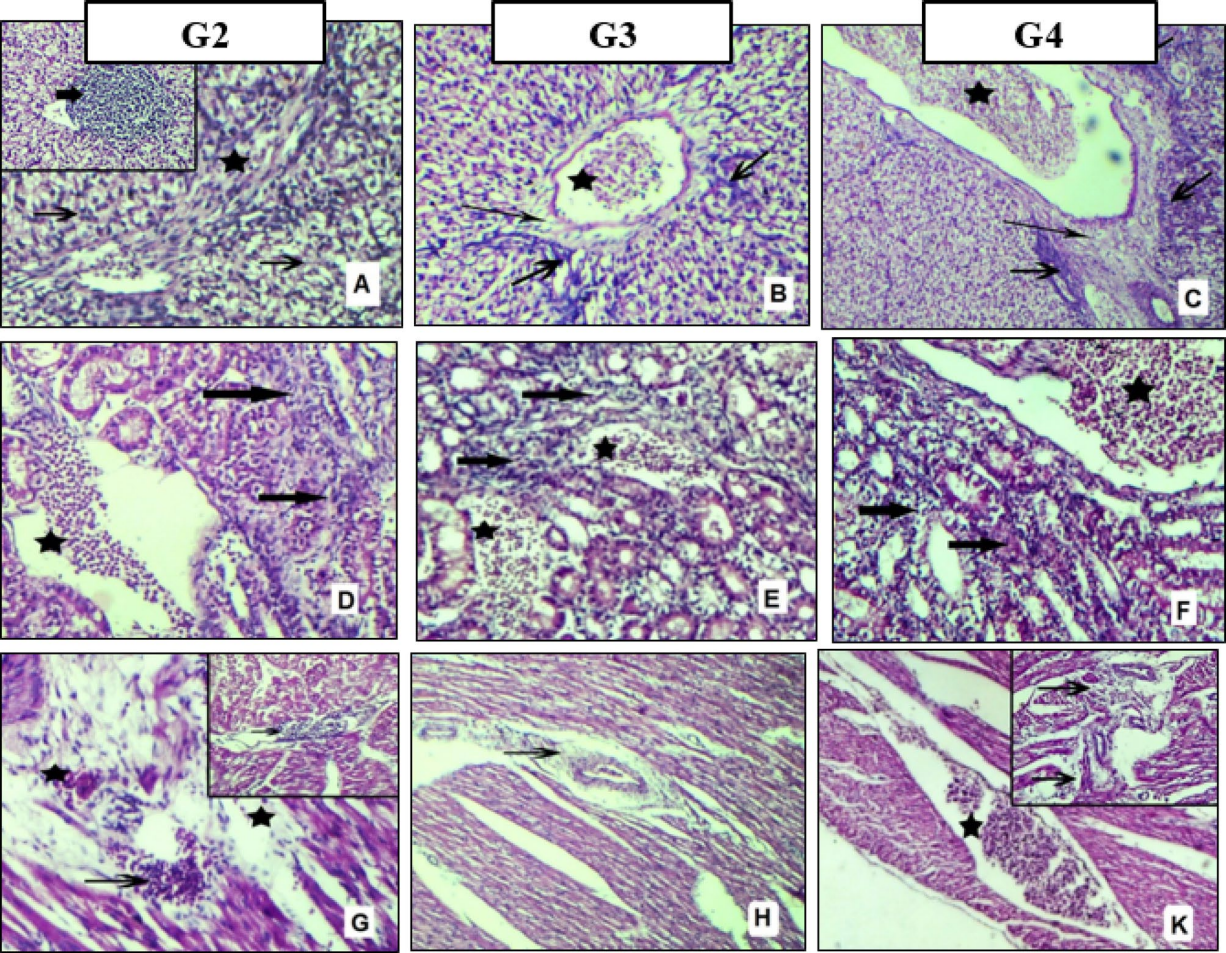
Pathological lesion of liver (**A**–**C**), kidney (**D**–**F**) and heart (**G**,**H**,**K**).

**Fig. 5:**
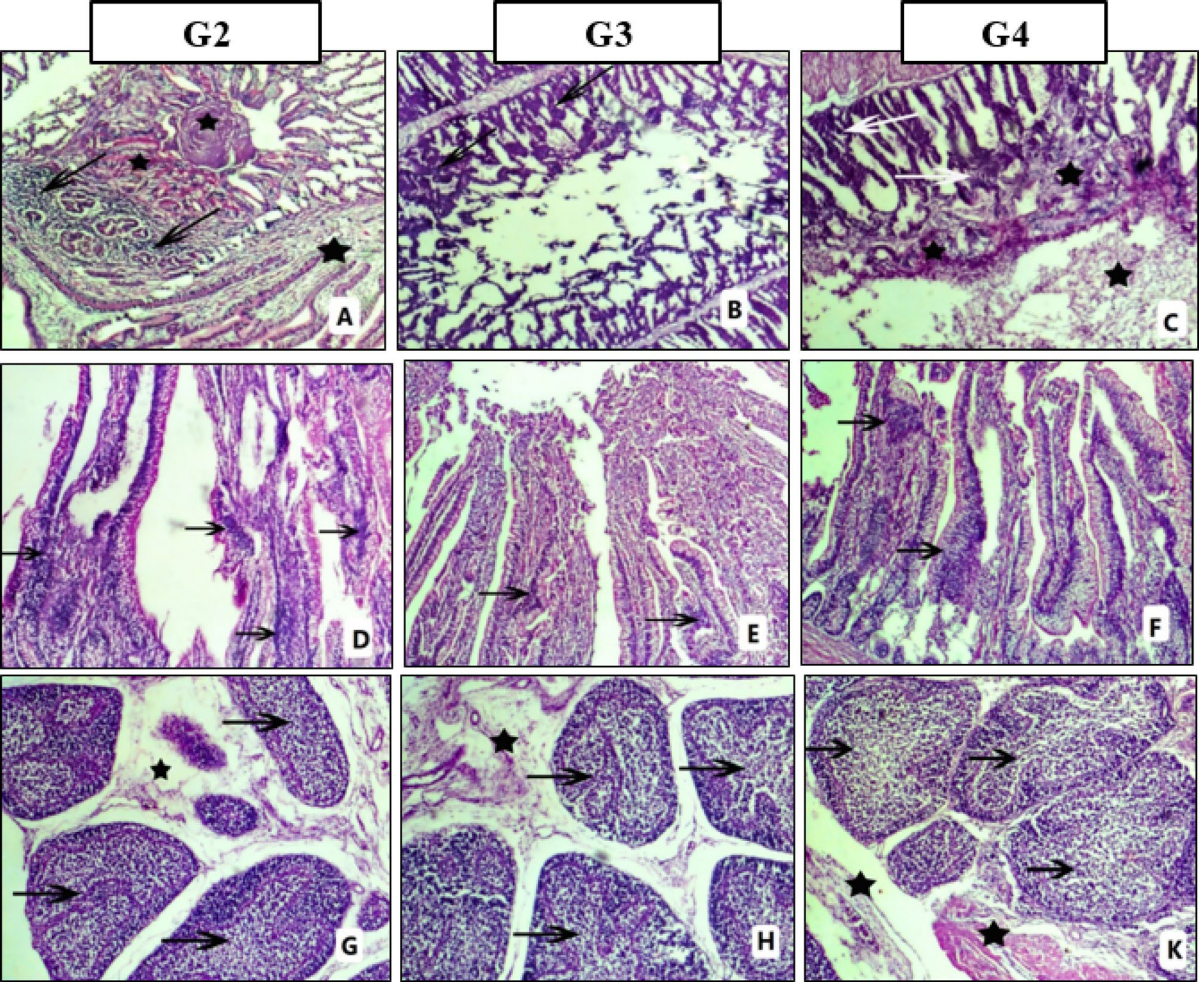
Pathological lesion of Proventriculus (**A**–**C**), Intestine (**D**–**F**) and Bursa (**G**,**H**,**K**).

As shown in Figs. 4 and 5, all samples collected from G1 revealed no lesions. The most lesions were more severe in G2 which was challenged with *Salmonella* only and less severe in G4 which was challenged and treated with specific antibiotic and mild lesions were observed in G3 which was challenged and treated with symbiotic. The severity of the lesions between different groups is shown in Table 11. The most pronounced lesions were vacuolar degeneration, necrosis, and inflammatory cell infiltration in addition to interstitial edema, hemorrhage, congestion in the liver, kidney, heart (Fig. 4), and proventriculus (Fig. 5A–C). The intestine showed variable degrees of degeneration, necrosis, and sloughing of the villus epithelium with inflammatory infiltration (Fig. 5D–F), while the cecum revealed cryptal necrosis and inflammatory cell aggregation. Also, in the bursa of fabricius there were follicular atrophy and lymphocytic depletion in addition to interfollicular edema (Fig. 5G, H,K). Also, lymphocytic depletion was seen in spleen, thymus, and tonsil in G2 and less severe in G3 & G4.

**Hepatocytes** showing: (A) vacuolar degeneration (thin arrow), necrosis with inflammatory cells infiltration (thick arrow), edema, hemorrhage and loss of liver cord appearance (asterisk), (B&C) congestion (asterisk), perivascular edema (thin arrow), vacuolar degeneration, necrosis infiltrated with inflammatory cells (thick arrow). **Kidney** showing variable degrees of tubular degeneration, necrosis with inflammatory cells infiltration (arrow), interstitial edema and hemorrhage (asterisk). **Myocardial muscl**e showing: (G) degeneration, necrosis with inflammatory cells infiltration (arrow), interstitial edema and hemorrhage (asterisk), (H) perivascular edema (arrow), (K) congestion (asterisk), edema, degeneration and necrosis infiltrated with mononuclear cell (arrow). **(H&E stain x100-200).**

**Proventriculus** showing variable degrees of edema and hemorrhages within and between the follicular spaces (asterisk), necrosis and devastation of glandular cells and mucosal folds with mononuclear cell infiltration (arrow). **Intestine** showing variable degrees of intestinal necrosis, atrophy, and sloughing of the villus infiltrated with inflammatory cells (arrow). **Bursa** showing variable degrees of follicular atrophy, lymphocytic depletion (arrow) and interstitial edema (asterisk). **(H&E stain x100).**

**Table 11:** Histopathological score lesions within different groups.

Organ	Lesion	G2	G3	G4
Liver	vacuolar degeneration	++	++	++
	focal necrosis and inflammatory infiltration	+++	+	++
	perivascular edema	+++	+	++
	Congestion	+	+	++
Kidney	tubular degeneration, necrosis and mononuclear cell infiltration	+++	+++	+++
	edema and hemorrhage in the interstitium	++	++	++
Heart	necrosis and mononuclear cell infiltration	+++	**-**	+++
	edema and hemorrhage in the interstitium	+++	+	+++
	Congestion	-	-	++
Proventriculus	necrosis and destruction of mucosal folds with inflammatory infiltration	+++	+	++
	edema and hemorrhages within and between the follicular space	+++	-	+++
Intestine	intestinal necrosis and sloughing of the villus epithelium with inflammatory infiltration	+++	+	++
Bursa	follicular atrophy	+++	+++	++
	lymphocytic depletion	+	++	+++
	interstitial edema	+++	+++	++

Appreviations: +++ = severe, ++ = moderate, + = mild and **-** = no lesions.

## Discussion

The current investigation detected *Salmonella* species on four farms out of twenty-five, which represents 16% (40 samples out of 250 samples). This result was nearly like to 17% and 16.7% as previously reported by Nabil and Younis^34^ and Abdi et al.^35^, respectively. Lower rates have been demonstrated in prior researches: 4% Alexan et al.^36^, 14.55% Chea et al.^37^ and 12.7% Saad et al.^38^. However, it was considerably less than 41% and 36.5% as documented by El Sharkawy et al.^39^ and Polat et al.^40^, respectively. This variation may be attributed to regional and seasonal variation, differences in the housing system, age, breeds, poultry densities, and farm biosecurity measures. The study identified multiple *Salmonella* serotypes represented in *S.* Papuana, *S.* Enteritidis, and *S.* Kentucky. *S.* Enteritidis represent the most frequent isolates which in line with Wang et al.^41^, Abd El-Mohsen et al.^42^ and Shen et al.^43^. Although *S.* Papuana is less frequently encountered than dominant strains like *S.* Enteritidis, it remains a notable concern within the poultry industry, due to its ability to persist throughout the production process, marking it as a significant emerging foodborne pathogen^44^.

Antibiotic resistance profiles have been demonstrated by *Salmonella* showed high resistance toward amoxicillin, lincomycin, and spiramycin (100% each) followed by ampicillin 75% (Table 4). nearly similar finding reported by Alexan et al.^36^] who reported resistance toward amoxicillin (100%), and Piryaei et al.^14^, who found (80.41%%) toward amoxicillin–clavulanate and (83.5%) against ampicillin. Also, Nabil et al.^45^, and Islam et al.^46^ reported resistance against AMP with 73.08%, and 75%, respectively. The noticed resistance toward *β*-lactam antibiotics (amoxicillin and ampicillin) may be because it is the most prescribed class of antibiotics Escamilla et al.^47^. *Salmonella* in the current study demonstrated 100% sensitivity to colistin sulfate, in contrast Alexan et al.^36^ recorded higher resistance 100%. MAR index values of isolated strains varied from 0.21 to 0.43, which indicates frequent use of antibiotics as recorded by Piryaei et al.^14^ suggesting poultry products as a source of MDR *Salmonella* strains.

The ability of *Salmonella* to infect hosts is enabled by different virulence factors. One of them is the *invA* gene, which is necessary to invade host epithelial cells. All examined serotypes harbored the *invA* gene (100%) (Fig. 1C), which was similar to previous studies Shen et al.^43^ and Kelly & Iang^48^. Also, all isolated strains contained the Heat-labile *Salmonella* enterotoxin gene *stn* (100%), which was considered an indicator for pathogenicity potential and public health risks (Fig. 1A). Shen^43^, Jafari Sohi et al.^49^ and Gharaibeh et al.^50^ recorded the presence of *stn* gene in 100%, 96.11% and 75.3% of *Salmonella* isolates, respectively. In this study 75% of *Salmonella* isolates carried the spvC gene (Fig. 1B). which is a virulent gene carried on the plasmid, encoding phosphor threonine lyase that is essential for survival within the host cell responsible for suppression of intestinal lesions^51^. Other studies showed higher prevalence 97.8% Gharaibeh et al.^50^ and 96.90% Piryaei et al.^14^], however a decreased frequency was detected by Eid et al.^52^ 0% and Ammar et al.^53^ 5.88%. It was noticed that gene prevalence varied by serotype, source, and region. The difference in prevalence of the *spv*C gene with other studies is due to its plasmid-borne nature, and is typically found in a limited number of serovars, mainly in *S*. Typhimurium, *S*. Enteritidis, and *S*. Dublin^54^. The predominance of *S*. Enteritidis, which conserved this plasmid, explains the high detection. Additionally^55^, explained the lower incidence owing to environmental factors or plasmid loss during sub-culturing and sample handling.

In this experiment synbiotics and antibiotics effectiveness in clinical symptoms and PM lesions of infected birds, reducing *S.* Enteritidis colonization in broiler chicks, immunity status, and associated pathological changes were evaluated. Regarding clinical symptoms, the infected birds showed anorexia, weight reduction, and abnormal breathing patterns, watery diarrhea with a whitish appearance, and occasional lameness. These signs were more prominent in G2 and decreased in severity in groups 3 & 4, respectively (Fig. 2). Same symptoms were detected by Nazir et al.^56^, Wibisono et al.^5^, and El-Sherry^57^.

The PM lesions of infected birds showed air sacculitis, severe congestion and increase in size of liver, spleen, and kidney with urates deposition in ureters. These lesions were more prominent in G2 and decreased in severity in groups 3 & 4, respectively (Fig. 3). The observed lesions were compatible with those of Kumari et al.^58^, Wibisono et al.^5^, and El-Sherry^57^, and nearly like that recorded by Nazir et al.^56^.

Concerning growth performance, the results of growth performance (Table 6) are similar to that noticed by Salag et al.^59^ and Sobotik et al.^26^. The benefits of synbiotic in G3 attributed to positive influence of its component (probiotic) in stimulation of beneficial microorganisms in the digestive system Awad et al.^60^ and Mookiah et al.^61^. Many results determined the role of synbiotics in competing harmful microorganism, motivate multiplication of useful microbiota, and maintain healthy gut, those necessary for the absorption of nutrients Cheng et al.^62^. The development in the performance parameters because of probiotics administration could be attributed to enhancing the bird’s appetite Nahashon et al.^63^, increasing efficiency of feed Cavit^64^ and Haj et al.^65^, generating digestive enzymes Saarela et al.^66^, along with the positive impact on the well-being of the birds Soomro et al.^67^.

Regarding cecal colonization, the slight decrease in *Salmonella* colonization in G3 compared to G4 (Table 7) may be due to the adverse impact of florfenicol on beneficial microbiota that help in elimination of pathogenic microorganisms. This finding agreed to some extent with Mei et al.^68^, who clarified that florfenicol primarily affects linoleic acid metabolism, which changes the intestinal microbiota and increases *Salmonella* infection, and Shang et al.^69^, who mentioned the role of antibiotics in reducing the accumulation of natural flora constituent species that help in eliminating unfavorable bacteria. The decrease in *Salmonella* colonization in symbiotic dietary supplemented group are consistent with those of Drauch et al.^70^ and Fayyaz et al.^71^, who observed marked reduction in cecal colonization of *S.* Infantis following synbiotic administration. Similarly, Olsen et al.^72^ and Kerek et al.^73^ reported a notable decrease in *S.* Enteritidis shedding in birds treated with probiotics compared with untreated birds. This decrease is due to natural bioactive substances in a synbiotic equilibrium that can modulate gut microbiota by enabling the digestive system to tolerate stimuli that are both infectious and non-infectious^74^ and stimulation of immunity through enhanced biliary anti-*Salmonella* IgA Luoma et al.^75^. In addition, probiotics, which are a component of synbiotics can shield the intestinal epithelium from pathogenic bacterial colonization, like *Salmonellosis* by limiting the penetration capacities of pathogens^76^ or suppress the multiply of bacteria through increasing the acidity of gut Al-Fatah^77^.

Hematological and biochemical metrics represent vital markers for health condition, nutrition, diseases, and physiological states of birds^78,79^. The results of hemogram (Table 8) align with those given by Sunu et al.^80^. Agboola^81^ stated that Hb is vital for life due to its delivery of oxygen to tissues. The increased Hb levels were due to digestive tract acidic media from prebiotic fermentation, enhancing iron absorption in the small intestine and vitamin B complex release by beneficial bacteria, potentially influencing erythropoiesis^82^.

Synbiotics may improve the digestibility and accessibility of numerous nutrient components, proteins, minerals, and vitamins^83^. The intake of these nutrients might raise Hb levels, indicating an enhancement in the nutritional, respiratory, and enzymatic functions of the blood and contributing to an increase in the body’s defenses. Generally, the decreased haemoglobin and PCV is associated with anaemia. Lower values of haemoglobin and packed cell volume in indigenous chicken also corresponded with the acute phase of disease in which anaemia had been reported in chickens^84,85^. ON the other hand, the results of leukogram (Table 8) are compatible with the results those reported by Unigwe et al.^86^. Elevated leukocyte production reflects the body’s immune response to infection^87^. According to Mohamed et al.^88^, heterophil percentages above 31.95% are indicative of physiological stress. Heterophils act as the chief defense mechanism against infections^89^. As a response to the *S.* Enteritidis challenge, these cells function as key components of the immune system, contributing to the protection of the birds^90^. An elevated basophil count in chicken blood indicates abnormal conditions, including stress caused by excessively high temperatures or pathogenic infections^91^.

Regarding to phagocytic activity and phagocytic index, the current results (Table 9) align with Borchers et al.^92^, who stated the impact of probiotics in enhancing the organism’s resistance by improving antibody levels and increasing macrophage efficiency, with the benefits of probiotic colonization being important for creating a functional immune system. It involves lymphocyte recruitment, IgA production, and the establishment of immune tolerance to encounter antigens.

The results of liver and kidney functions (Table 10) are similar to those noticed by Okuneye et al.^93^ and Unigwe et al.^86^. ALT and AST are serum liver enzymes, typically not detected in the blood unless liver cells are harmed. Thus, these enzymes are employed to estimate liver health through its functional ability, cellular integrity, and the status of the biliary system^94,95^. Significant elevation of ALT is because infection with *S.* Enteritidis can cause this^96^. While principally an intestinal pathogen, *S.* Enteritidis can enter the mucosal barrier, causing systemic spread and the establishment of localized infection sites, involving hepatic abscesses^97^. Regarding serum proteins, globulin concentrations act as a crucial indicator of immune response and antibody production^98^. The levels of serum albumin and globulin depend on the availability of protein in the diet^99^. Reduced total protein levels, mainly in globulin, will lead to hypoproteinemia overall and specifically hypoglobulinemia^100^. This will result in a decrease in immunoglobulin (antibody) levels, producing a weak immune response^101^, which will lead to bird mortality^101,102^.

Uric acid, BUN, and creatinine are indicators for assessment of kidney’s function^103^. The level of serum uric acid displays renal function in chickens. Uric acid (UA) is the primary nitrogenous waste in birds and is excreted in feces (Sturkie^104^). Birds exhibiting lower uric acid levels indicate effective protein use and decreased deamination^105^. On the other hand, high serum uric acid levels often indicate the intake of diets containing unbalanced amino acids, leading to ineffective protein utilization^106^.

Concerning antioxidant enzymes, our results agree with those obtained by Song et al.^107^. Activity variation of antioxidant enzymes in addition to the depletion of certain nonenzymatic antioxidants are the most prevalent indicators of oxidative stress^108^. Excessive production of reactive oxygen species (ROS) and nitrogen, coupled with oxidative stress, is a major factor contributing to cellular damage and reduced performance in chickens^109^. Synbiotics enhance the removal of ROS by stimulating antioxidant enzymes and strengthen the overall antioxidant effect^110^.

Pathogenesis of *Salmonella* involves multiple stages: adhesion, penetration of intestinal epithelium, survival, multiplication, and extraintestinal dissemination^111^. *Salmonella* can pass through the acidic stomach environment due to its ability to withstand 3.7 pH, and upon reaching the small intestine, *Salmonella* utilizes fimbrial adhesins to attach to and colonize the intestinal epithelium, then penetrates the intestinal mucosa via M cells of the Peyer’s patches, which carry *Salmonella* to underlying lymphoid follicles, like mononuclear cells and macrophages^112^.

Macrophages can engulf *Salmonella* but are unable to destroy it, as *Salmonella* prevent*s* phagosomes from fusing with secondary lysosomes, which is how macrophages eliminate intracellular pathogens, resulting in intracellular withstand of bacteria. *Salmonella* survives and replicates inside macrophages, which facilitate its spread to mesenteric lymph nodes and then to systemic organs such as the liver, heart, and spleen^113^. This explains our pathological lesions, which were degeneration, necrosis, and inflammatory cell infiltration in addition to interstitial edema and hemorrhage in all groups challenged with *Salmonella*, especially in the gastrointestinal tract, heart, and kidney. Also, there was lymphocytic depletion in the lymphoid organs, which revealed systemic infection (Figs. 4 and 5). These results matched Mohamad et al.^114^, Muna et al.^115^, and Hamed et al.^116^.

Pathological lesions were less severe in G4, which received antibiotics, and were mild in G3, which received synbiotics (Table 11). These findings are consistent with the reports of Stanley et al.^117^ and Zuamí et al.^118^; they explain the enhancement effect of synbiotic supplementation on the integrity and functionality of broiler intestinal mucosa, which enables broilers to fend off *Salmonella* infections, also inhibits *Salmonella* replication, and decreases the severity and development of pathological lesions caused by subclinical necrosis, but disagree with Leticia et al.^119^, who found no impact on *Salmonella* infection in broilers or laying hens.

In spite of the sensitivity of florfenicol in the treatment of *Salmonella*, it has an adverse effect on gut microbiota. Mei^68^ attributed this to the decrease in the level of CLA (conjugated linoleic acid) that is formed by lactobacillus and the increase in the level of diHOME. The role of CLA is to prevent *Salmonella* multiplication, decrease inflammation, and preserve intestinal health, while diHOME compounds promote pro-inflammatory effects on the intestinal epithelium and vascular endothelial cells; this explains the milder effect of florfenicol than synbiotic on *Salmonella* infection.

## Conclusion

This study could validate synbiotics as a safe renewable replacement for antibiotics in the poultry sector. This approach not only improves animal welfare and productivity through improvement of growth, hematological and immunological parameters, but also reduces the risk of multidrug-resistant *Salmonella.*

## Data Availability

The datasets used and/or analyzed during the current study are available from the corresponding author on reasonable request.

## Declarations

### Guidelines

All methods were carried out in accordance with relevant guidelines and regulations.

### ARRIVE guidelines

The authors confirm that the study was carried out in compliance with the ARRIVE guidelines.
